# Family Disadvantage, Education, and Health Outcomes Among Black Youths Over a 20-Year Period

**DOI:** 10.1001/jamanetworkopen.2024.2289

**Published:** 2024-03-29

**Authors:** Edith Chen, Tianyi Yu, Katherine B. Ehrlich, Phoebe H. Lam, Tao Jiang, Thomas W. McDade, Gregory E. Miller, Gene H. Brody

**Affiliations:** 1 Institute for Policy Research, Northwestern University, Evanston, Illinois; 2 Department of Psychology, Northwestern University, Evanston, Illinois; 3 Center for Family Research, University of Georgia, Athens; 4 Department of Psychology, University of Georgia, Athens; 5 Department of Psychology, Carnegie Mellon University, Pittsburgh, Pennsylvania; 6 Department of Anthropology, Northwestern University, Evanston, Illinois

## Abstract

**Importance:**

Upward mobility (via educational attainment) is highly valued, but longitudinal associations with mental and physical health among Black youths are less understood.

**Objective:**

To examine associations of childhood family disadvantage and college graduation with adult mental and physical health in Black youths followed up into adulthood.

**Design, Setting, and Participants:**

This longitudinal, prospective cohort study of Black youths from the state of Georgia who were studied for 20 years (ages 11 to 31 years) was conducted between 2001 and 2022. Participants for this study were drawn from the Strong African American Healthy Adults Program. Data analysis was conducted from April 2023 to January 2024.

**Exposures:**

Family economic disadvantage (measured during the adolescent years) and college graduation (indicating upward mobility).

**Main Outcomes and Measures:**

Primary outcomes included mental health, substance use, and physical health. Mental health included a composite of internalizing and disruptive problems (anxiety, depression, anger, aggressive behaviors, and emotional reactivity). Substance use included a composite of smoking, drinking, and drug use. Physical health included metabolic syndrome (MetS) and proinflammatory phenotypes (immune cells mounting exaggerated cytokine responses to bacterial challenge and being insensitive to inhibitory signals from glucocorticoids). Mental and physical health measures were taken at age 31 and during the adolescent years. Linear and logistic regression analyses, as well as mediated moderation analyses, were conducted.

**Results:**

The study population consisted of 329 Black youths (212 women [64%]; 117 men [36%]; mean [SD] age at follow-up, 31 [1] years). Compared with those who did not graduate college, those who graduated from college had 0.14 SD fewer mental health problems (*b* = −1.377; 95% CI, −2.529 to −0.226; β = −0.137; *P* = .02) and 0.13 SD lower levels of substance use (*b* = −0.114; 95% CI, −0.210 to −0.018; β = −0.131; *P* = .02). Residualized change scores revealed that college graduates showed greater decreases from age 16 to 31 years in mental health problems (*b* = −1.267; 95% CI, −2.360 to −0.174; β = −0.133; *P* = .02) and substance use problems (*b* = −0.116; 95% CI, −0.211 to −0.021; β = −0.136; *P* = .02). For physical health, significant interactions between childhood family disadvantage and college completion emerged in association with MetS (OR, 1.495; 95% CI, 1.111-2.012; *P* = .008) and proinflammatory phenotype (*b* = 0.051; 95% CI, 0.003 to 0.099; β = 0.131; *P* = .04). Among youths growing up in disadvantaged households, college completion was associated with a 32.6% greater likelihood of MetS (OR, 3.947; 95% CI, 1.003-15.502; *P* = .049) and 0.59 SD more proinflammatory phenotype (mean difference, 0.249, 95% CI, 0.001 to 0.497; *P* = .049). Conversely, among those from economically advantaged backgrounds, college completion was correlated with lower MetS and less proinflammatory phenotype. Findings held after controlling for body mass index at age 19 years.

**Conclusions and Relevance:**

In this longitudinal cohort study of Black youths, graduating from college was associated with an adult profile of better mental health but poorer physical health among those from economic disadvantage. These findings suggest that developing interventions that foster healthy outcomes across multiple life domains may be important for ensuring that striving for upward mobility is not accompanied by unintended cardiometabolic risk.

## Key Points

**Question:**

Are there associations of childhood disadvantage and educational attainment with mental and physical health among Black youths?

**Findings:**

In this cohort study of 329 Black youths, those who grew up in economically disadvantaged households and graduated college displayed an adult profile of better mental health and less substance use but also a proinflammatory phenotype and greater likelihood of metabolic syndrome compared with those not graduating. College graduation was associated with better mental and physical health among those from economically advantaged households.

**Meaning:**

These findings suggest that understanding the health outcomes of college graduates across racial and income groups is important for developing interventions to maximize the benefits of striving for upward mobility.

## Teaser

This longitudinal cohort study of Black youths assesses associations of economic disadvantage and educational attainment in childhood and adolescence with physical and mental health outcomes in adulthood.

## Introduction

Upward mobility (eg, educational achievement) is viewed as desirable, opening doors to occupational and financial opportunities throughout life that will be beneficial to well-being, health, and happiness.^1,2,3^ However, research^4,5,6^ indicates that not everyone benefits equally from upward mobility; there are diminishing returns (ie, fewer benefits for a given educational or income level) for Black and Hispanic individuals compared with White individuals. Moreover, upward mobility may even sometimes come at a cost to physical health.^7,8,9^

For example, in 2 US national samples^10^ with wide age ranges and varying races and ethnicities, adults were divided into life-course, socioeconomic-status (SES) trajectories based on income indicators. Those characterized by upward mobility (low childhood SES and high adult SES) had low levels of adult depression similar to those with high SES throughout their lives (high childhood SES and high adult SES group [high-high]). At the same time, however, this upwardly mobile group had higher rates of adult metabolic syndrome (MetS) than the high-high group.^10^ In another study,^11^ among Black and Hispanic individuals from low-SES backgrounds, completing college was associated with fewer adult depressive symptoms but a higher risk of MetS, whereas the same patterns were not found in White individuals. Additional evidence comes from analyses of low-SES families in France^12^ after the required years of schooling was increased; adult blood pressure was higher in those born after the school mandate was implemented compared with those born before. This natural experiment^12^ suggests that increasing years of schooling for those with low SES could have physical health costs. Taken together, these results suggest a pattern termed *skin-deep resilience*, whereby above the skin, upwardly mobile youths achieve successes by many external metrics (going to college, earning higher incomes, and having good mental health); however, below the skin, they appear to be struggling in terms of cardiometabolic health, suggesting resilience that is only skin-deep.^7,8^

These patterns are thought to exist because, for many individuals experiencing disadvantage, the path to success is paved with multiple challenges and hardships (eg, lack of resources, interpersonal discrimination, and structural racism). In turn, such stressors experienced earlier in life are associated with dysregulation of key biological processes, such as inflammation, which contribute to risk for cardiometabolic diseases in adult life.^13,14^

The present study reports on a 20-year longitudinal investigation focused on Black youths, given the excess mortality in this population^15^ and given the importance of understanding the origins of health disparities by studying precursors to chronic diseases earlier in life.^16^ This study tests the skin-deep resilience hypothesis, considering associations of upward mobility (educational attainment) with mental and physical health in adulthood. Mental health (internalizing symptoms and disruptive behaviors) and substance use outcomes were measured. Physical health outcomes included metabolic syndrome and inflammatory mechanisms, given the key role that inflammation plays in numerous chronic diseases of aging,^17,18^ and the importance of identifying early warning signs (prior to disease onset) in younger people.^13,19^ In line with skin-deep resilience theory, Black adolescents from low-SES households who graduated from college were hypothesized to have better adult mental health but poorer physical health compared with those without the same level of educational attainment or those with high SES.

## Methods

This longitudinal cohort study, spanning 2001 to 2022, was drawn from the Strong African American Healthy Adults Program (SHAPE).^8^ The University of Georgia’s institutional review board approved the protocol. Written consent was obtained from participants and caregivers. The study followed the Strengthening the Reporting of Observational Studies in Epidemiology (STROBE) reporting guideline. See the eMethods in Supplement 1 for more details.

### Participants

In 2001, SHAPE enrolled 667 Black children in fifth grade and a primary caregiver from 9 rural counties in the US state of Georgia. Caregivers identified their children’s race as Black, and families, on average, were characterized as working poor.^8^ From 2009 to 2010 (age 19 years), a subgroup of 500 participants was randomly selected for a substudy of stress and health. From 2021 to 2022 (age 31 years), the substudy cohort was reassessed and blood samples were obtained from 346 participants.

### Procedures

Data were collected primarily in participants’ homes. A phlebotomist drew fasting blood and collected health measures. See the eMethods in Supplement 1 for more details.

### Measures 

#### Family Disadvantage (Ages 16-18 Years)

Parents completed a measure of Unmet Material Needs.^20^ Three waves of assessments (ages 16, 17, and 18 years) were averaged to form a mean family unmet needs composite.

#### Bachelor’s Degree Completion

At age 31 years, participants reported whether they had completed a bachelor’s degree. Very few of the participants’ caregivers had bachelor’s degrees (18 of 329 caregivers [5.5%]), hence the use of this measure as an indicator of upward mobility.

#### Mental Health Composite (Ages 16 and 31 Years)

Mental health measures at age 31 covered both internalizing and disruptive problems, including anxiety,^21^ depression,^22^ anger,^21^ aggressive behaviors,^23^ and emotional reactivity.^24^ Measures were standardized and summed to form an adult mental health composite.

Youth mental health measures were taken at age 16 years. Standardized mental health measures often differ in childhood vs adulthood, and, thus, this longitudinal study contained different measures at ages 16 and 31 years. Nonetheless, at age 16 years, comparable constructs were assessed for anxiety,^25^ depression,^26^ anger,^27^ aggressive behaviors,^25^ and impulsivity.^28^ Measures were standardized and summed to form an age 16 mental health composite.

#### Substance Use Composite (Ages 16 and 31 Years)

At ages 16 and 31 years, participants reported their past-month cigarette, alcohol, and marijuana use (items from the Monitoring the Future Study^29^). Responses were summed to form a substance use composite consistent with prior research.^30^ Composites were log-transformed because of skewed distribution.

#### Metabolic Syndrome Diagnosis (Age 31 Years)

Glucose, high-density lipoproteins, and triglycerides were measured in fasting serum. Resting blood pressure and waist circumference were measured. MetS was diagnosed via the International Diabetes Federation guidelines.^31^

#### Proinflammatory Phenotype (Age 31 Years)

Inflammation, particularly when ongoing and dysregulated, is a key pathway to the pathogenesis of many chronic degenerative diseases.^17,18,32^ Because this study involved a longitudinal investigation of initially healthy Black children, measures of inflammatory processes were included as a potential early warning sign of chronic diseases later in life. Mechanistically, experiences of stress earlier in life are known to calibrate the response tendencies of innate immune cells so they mount excessive inflammatory responses to microbial challenges and become insensitive to glucocorticoid hormones that regulate these responses.^13,19^ This response tendency has been termed a proinflammatory phenotype.^13^

To assess proinflammatory phenotypes, we used a portable cell culture protocol developed for field settings,^33^ where immune cells are incubated ex vivo with a bacterial product (lipopolysaccharide [LPS]), and production of the proinflammatory cytokines interleukin-1β, interleukin-6, and tumor necrosis factor-α were measured. Hydrocortisone (3 concentrations) was added to separate cultures alongside LPS; because hydrocortisone inhibits cytokine production, this measures how sensitive cells are to the anti-inflammatory properties of glucocorticoids.

Analyses were conducted on 2 end points. The first was a stimulated cytokine production composite, formed by calculating a mean of the *z* scores of the 3 cytokines in the LPS well. Higher values represent larger cytokine responses to the bacterial product LPS. The second end point was a sensitivity to glucocorticoid inhibition composite, formed by estimating participant-specific sensitivity slopes for each cytokine at each hydrocortisone dose.^19,34^ The mean of the *z*-scored values of slopes was calculated. Higher values represent greater sensitivity to the anti-inflammatory properties of glucocorticoids.

#### Body Mass Index (Age 19 Years)

Because this study did not begin as a health study, MetS and proinflammatory phenotypes were not measured during childhood. To represent earlier physical health, at age 19 years, height and weight were assessed and body mass index (BMI) was calculated as weight in kilograms divided by height in meters squared.

#### Covariates

Child gender was included as a covariate. The SHAPE cohort^8^ was initially recruited for a randomized clinical trial to prevent behavior problems and substance use. Participation in the intervention was included as a covariate.

### Statistical Analysis

Study hypotheses were tested using linear regression equations (logistic regression for MetS) with sequentially entered blocks of variables including (1) covariates, (2) unmet material needs and bachelor’s degree completion, and (3) the 2-way interaction of these variables. Outcome variables included mental health, substance use, proinflammatory phenotype, and MetS at age 31 years. Interaction analyses were conducted according to established guidelines.^35^

Given the longitudinal nature of the study, we tested whether there were associations with change in mental health and substance use outcomes from ages 16 to 31 years using residualized change scores. For physical health outcomes, we statically controlled for BMI at age 19 years in analyses with health at age 31 years. Mediated moderation analyses were conducted using a regression-based procedure.

All statistical tests were 2-tailed with alpha = .05 and *P* < .05 as statistically significant. Analyses were conducted using SPSS statistical software version 29.0 (IBM). Data analysis was conducted from April 2023 to January 2024. See the eMethods in Supplement 1 for additional details.

## Results

The analytic sample consisted of 329 Black individuals (212 women [64%]; 117 men [36%]; mean [SD] age at follow-up, 31 [1] years) with complete data. The analytic sample did not differ from the sample of 500 participants from 2009 to 2010 who were missing data, except for being more likely to be a woman (eTable in Supplement 1). Of the 329 participants, 66 (20%) had completed a bachelor’s degree, and 125 (38%) met criteria for MetS diagnosis. Table 1 presents bivariate correlations. Compared with women, men had higher substance use, higher stimulated cytokine production, but lower BMI, and fewer MetS diagnoses. Family unmet needs were associated with more mental health problems (at age 16 years) and lower bachelor’s degree completion rates. Bachelor’s degree completion was associated with fewer mental health problems and less substance use (at age 31 years). Mental health and substance use problems were positively correlated. MetS and BMI were positively associated; proinflammatory phenotype and MetS were not correlated.

**Table 1.** Correlations Among Study Variables

Variable	Correlation coefficient^a^										
	Gender: man	Intervention	Mental health composite (age 16 y)	Substance use composite (age 16 y)	Unmet material needs (age 16-18 y)	BMI (age 19 y)	Bachelor’s degree completion	Stimulated cytokine production (age 31 y)	Sensitivity to AIS (age 31 y)	MetS diagnosis (age 31 y)	Mental health composite (age 31)
Gender: man	NA	NA	NA	NA	NA	NA	NA	NA	NA	NA	NA
Intervention	−.008	NA	NA	NA	NA	NA	NA	NA	NA	NA	NA
Mental health composite (age 16 y)	−.021	.022	NA	NA	NA	NA	NA	NA	NA	NA	NA
Substance use composite (age 16 y)	.153^b^	−.008	.282^c^	NA	NA	NA	NA	NA	NA	NA	NA
Unmet material needs (ages 16-18 y)	−.026	.054	.151^b^	.106	NA	NA	NA	NA	NA	NA	NA
BMI (age 19)	−.183^c^	−.017	.019	−.082	.064	NA	NA	NA	NA	NA	NA
Bachelor’s degree completion	−.087	−.042	−.078	−.028	−.167^c^	−.010	NA	NA	NA	NA	NA
Stimulated cytokine production (age 31 y)	.207^c^	−.051	.074	.043	.062	.014	.020	NA	NA	NA	NA
Sensitivity to AIS (age 31 y)	−.054	−.057	−.065	−.053	−.010	−.042	.014	−.179^b^	NA	NA	NA
MetS diagnosis (age 31y)	−.124^d^	−.055	.107	.004	.080	.340^c^	−.064	.007	−.027	NA	NA
Mental health composite (age 31 y)	−.086	.061	.317^c^	.081	.013	.049	−.111^b^	−.015	−.027	.090	NA
Substance use composite (age 31 y)	.252^c^	.028	.110^d^	.188^c^	.047	.015	−.124^d^	.034	−.043	−.024	.288^c^

Abbreviations: AIS, anti-inflammatory signaling; BMI, body mass index; MetS, metabolic syndrome; NA, not applicable.

^a^ Pearson correlations were presented for continuous variables and Spearman correlations were presented for dichotomous variables.

^b^ *P* < .01.

^c^ *P* < .001.

^d^ *P* < .05.

### Mental Health and Substance Use Composites

Compared with youths who did not complete a bachelor’s degree, those with a bachelor’s degree reported 0.14 SD fewer mental health problems at age 31 years (*b* = −1.377; 95% CI, −2.529 to −0.226; β = −0.137; *P* = .02) (Table 2). Compared with youths who did not complete a bachelor’s degree, those with a bachelor’s degree reported 0.13 SD less substance use at age 31 years (*b* = −0.114; 95% CI, −0.210 to −0.018; β = −0.131; *P* = .02) (Table 2).

**Table 2.** Childhood Family Unmet Material Needs and Bachelor’s Degree Completion and Their Association With Mental Health and Substance Use at Age 31 Years and Changes in Mental Health and Substance Use From Age 16 to 31 Years^a^

Factor	Mental health composite		Change in mental health composite		Substance use		Change in substance use	
	*b *(95% CI)^b^	β^c^	*b *(95% CI)^b^	β^c^	*b *(95% CI)^b^	β^c^	*b *(95% CI)^b^	β^c^
Gender: man	−0.498 (−1.415 to 0.418)	−0.059	−0.391 (−1.260 to 0.479)	−0.049	0.191 (0.114 to 0.267)^d^	0.262	0.169 (0.093 to 0.245)^d^	0.236
Intervention	0.565 (−0.320 to 1.451)	0.069	0.501 (−0.339 to 1.341)	0.065	0.015 (−0.059 to 0.089)	0.021	0.014 (−0.059 to 0.087)	0.020
Unmet material needs (ages 16-18 y)	0.003 (−0.184 to 0.190)	0.002	−0.055 (−0.232 to 0.122)	−0.037	0.009 (−0.006 to 0.025)	0.070	0.006 (−0.009 to 0.022)	0.047
Bachelor’s degree completion	−1.377 (−2.529 to −0.226)^e^	−0.137	−1.267 (−2.360 to −0.174)^e^	−0.133	−0.114 (−0.210 to −0.018)^e^	−0.131	−0.116 (−0.211 to −0.021)^e^	−0.136
Unmet material needs × bachelor’s degree completion	−0.162 (−0.623 to 0.300)	−0.044	−0.224 (−0.663 to 0.214)	−0.064	−0.025 (−0.063 to 0.014)	−0.077	−0.022 (−0.061 to 0.016)	−0.071

^a^ Based on a total of 329 participants.

^b^ Unstandardized regression coefficient.

^c^ Standardized regression coefficient.

^d^ *P* < .001.

^e^ *P* < .05.

### MetS

There was a significant interaction between unmet material needs and bachelor’s degree completion associated with MetS (odds ratio [OR], 1.495; 95% CI, 1.111-2.012; *P* = .008) (Table 3 and Figure). Below we report comparisons of those with and without a bachelor’s degree among participants who grew up with low unmet material needs (1.5 SD below the mean) and those with high unmet needs (1.5 SD above the mean).

**Table 3.** Childhood Family Unmet Material Needs and Bachelor’s Degree Completion and Their Association With Physical Health Outcomes at Age 31 Years^a^

Factors		Stimulated cytokine production		Sensitivity to anti-inflammatory signaling		Metabolic syndrome diagnosis, OR (95% CI)
		*b *(95% CI)^b^	β^c^	*b *(95% CI)^b^	β^c^	
Gender: man		0.168 (0.073 to 0.263)^d^	0.189	−0.004 (−0.022 to 0.014)	−0.025	0.532 (0.325 to 0.870)^e^
Intervention		−0.048 (−0.140 to 0.044)	−0.055	−0.008 (−0.025 to 0.009)	−0.050	0.752 (0.473 to 1.195)
Unmet material needs (ages 16-18 y)		0.004 (−0.015 to 0.024)	0.025	0.001 (−0.002 to 0.005)	0.044	0.998 (0.906 to 1.098)
Bachelor’s degree completion	0.051 (−0.068 to 0.171)		0.048	−0.002 (−0.024 to 0.020)	−0.010	1.216 (0.655 to 2.255)
Unmet material needs × bachelor’s degree completion	0.051 (0.003 to 0.099)^e^		0.131	−0.009 (−0.018 to 0.000)	−0.125	1.495 (1.111 to 2.012)^f^

Abbreviation: OR, odds ratio.

^a^ Based on a total of 329 participants.

^b^ Unstandardized regression coefficient.

^c^ Standardized regression coefficient.

^d^ *P* < .001.

^e^ *P* < .05.

^f^ *P* < .01.

**Figure.**
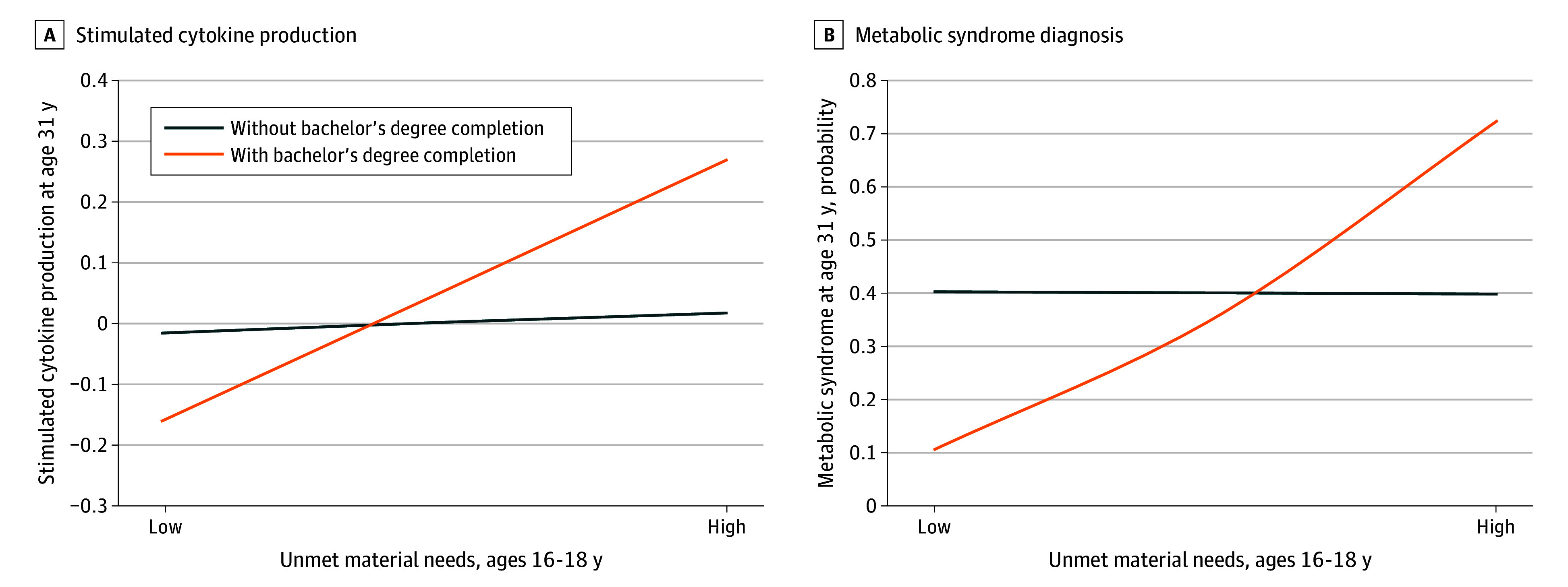
Childhood Family Unmet Material Needs, Bachelor’s Degree Completion, and Health Outcomes The figure shows stimulated cytokine production (A) and metabolic syndrome diagnosis (B) at age 31 years as a function of childhood family disadvantage (family’s unmet material needs at ages 16-18 years) and bachelor’s degree completion.

When participants experienced little financial hardship (low unmet material needs) as teenagers, those who completed their bachelor’s degree had a 29.6% lower risk of adult MetS compared with those without a bachelor’s degree (OR, 0.171; 95% CI, 0.048-0.608; *P* = .006). In contrast, when participants experienced high financial hardship as teenagers, those who completed their bachelor’s degree had a 32.6% higher risk of adult MetS compared with those without a degree (OR, 3.947; 95% CI, 1.003-15.502; *P* = .049).

### Proinflammatory Phenotype

There was a significant interaction between unmet material needs and bachelor’s degree completion for stimulated cytokine production (*b* = 0.051 [95% CI, 0.003 to 0.099]; β = 0.131; *P* = .04) but no significant interaction for sensitivity to glucocorticoids (b = −0.009; 95% CI, −0.018 to 0.001; β = −0.125; *P* = .05) (Table 3 and Figure).

When participants experienced little financial hardship as teenagers, those with a bachelor’s degree had cells with 0.42 SD higher sensitivity to glucocorticoids compared with those without a degree, although the result was not statistically significant (mean difference, 0.033; 95% CI, −0.003 to 0.069; *P* = .07). No differences were found for LPS-stimulated cytokine production (mean difference, 0.147; 95% CI, −0.341 to 0.047; *P* = .14).

In contrast, when participants experienced high financial hardship as teenagers, those who earned a bachelor’s degree had a 0.59 SD higher level of LPS-stimulated cytokine production compared with those without a degree (mean difference, 0.249, 95% CI, 0.001 to 0.497; *P* = .049). No differences were found for sensitivity to glucocorticoids (mean difference, −0.037; 95% CI, −0.083 to 0.009; *P* = .12).

### Earlier Health Measures

We tested whether the results held when using residualized change scores from age 16 to 31 years (Table 2). Compared with those without a bachelor’s degree, those with a bachelor’s degree displayed a 0.13 SD decrease in mental health problems (*b* = −1.267; 95% CI, −2.360 to −0.174; β = −0.133; *P* = .02) and a 0.14 SD decrease in substance use from age 16 to 31 years (*b* = −0.116; 95% CI, −0.211 to −0.021; β = −0.136; *P* = .02).

After controlling for BMI at age 19 years, the interactions at age 31 years between family unmet material needs and bachelor’s degree completion remained for MetS (OR, 1.441; 95% CI, 1.061 to 1.956; *P* = .02) and measures of proinflammatory phenotype including LPS-stimulated cytokine production (*b* = 0.049; 95% CI 0.001 to 0.098; β = 0.127; *P* = .04) and sensitivity to glucocorticoids (b = −0.009; 95% CI, −0.017 to 0.000; β = −0.121; *P* = .06).

### Additional Analyses: Moderation and Mediated Moderation

No significant moderation by participant gender or intervention status was found (race and age were the same in all participants and not tested as moderators). We tested whether stimulated cytokine production or sensitivity to glucocorticoids mediated the interaction between unmet material needs and bachelor’s degree completion or MetS. Neither stimulated cytokine production (OR, 0.948; 95% CI, 0.541 to 1.662; *P* = .85) nor sensitivity to glucocorticoids (OR, 0.645; 95% CI, 0.034 to 12.393; *P* = .77) was significantly associated with MetS. The indirect effects of the unmet material needs and bachelor’s degree interaction with MetS were not significant for LPS-stimulated cytokine production (indirect effect, −0.003; 95% CI, −0.053 to 0.031) or sensitivity to glucocorticoids (indirect effect, 0.004; 95% CI, −0.026 to 0.035).

## Discussion

In this 20-year longitudinal cohort study of Black youths, upward mobility (defined as completing one’s bachelor’s degree) was associated with a skin-deep resilience profile of good mental health but worse physical health in adulthood, particularly among those growing up in low-SES households. That is, among Black teenagers living under economic disadvantage, going on to complete college was associated with better mental health profiles and less substance use, but also, a greater likelihood of MetS diagnosis and indications of a more pronounced proinflammatory phenotype in adulthood. Conversely, among Black teenagers from high-SES households, completing college was associated with good mental and physical health at age 31 years. Change analyses demonstrated that college completion was associated with greater decreases from age 16 to 31 years in mental health problems and substance use in all youths. Findings with MetS and proinflammatory phenotypes held after controlling for earlier BMI. These results suggest that striving for upward mobility, while associated with beneficial life outcomes such as good mental health, may at the same time set Black youths from low-SES households on a trajectory to dysregulated inflammatory signaling and cardiometabolic risk in adulthood.

Our findings are consistent with other studies^10,11^ of upward mobility and mental vs physical health. The present study extends previous research by seeking to understand patterns solely within a Black population (other studies of upward mobility and mental and physical health contained approximately 60%-80% White participants^10,11^). The present study also focused on educational mobility in contrast with previous studies of income mobility.^10^ In addition, the present study conducted a longer follow-up (20 years) than any previous skin-deep resilience study and accounted for earlier mental health and physical health, which was not possible in previous studies.^10,36^

There are numerous possible explanations for the study findings. One is that individuals seeking upward mobility frequently exert high levels of persistent striving in order to succeed. Such striving and hard work facilitate life successes; in turn, successes may be beneficial to mental health. At the same time, however, prolonged striving and hard work can be exhausting and may take a cumulative physiological toll on physical health.^8,37,38,39,40,41^ A second explanation is that when faced with stress, particularly in environments with substantial constraints, some individuals may turn to coping strategies that help alleviate distress but are detrimental to physical health (eg, overeating of comfort foods).^42,43,44^ Other explanations for why achieving higher SES might be associated with worse physical health among Black individuals include the increased discrimination experienced when individuals move into predominantly White spaces for higher education or work, the increased social isolation from the Black community,^4,45,46,47^ and structural racism (eg, in employment opportunities and housing) that hinders the ability of Black individuals to take advantage of resources that supposedly come with moving into higher SES brackets.^4,47,48^

Evidence for skin-deep resilience was found both with MetS and immunologically in terms of cells exhibiting larger cytokine responses to bacterial challenge. Previous skin-deep resilience research assessed inflammatory biomarkers such as C-reactive protein,^49^ but these markers are not necessarily a causal part of mechanistic pathways involving inflammation because they can sometimes be released in response to other signals without underlying injury or infection.^50^ Thus, to our knowledge, this study provides the first empirical evidence in healthy young people of skin-deep resilience–related alterations to key processes in how immune cells function (eg, how aggressively cells produce cytokines in the face of bacterial challenges). Black individuals from low-SES households achieving upward mobility were prone to exhibiting a proinflammatory phenotype in their adult years. They also were more likely to experience clinical diagnoses such as MetS. However, no evidence for mediated moderation emerged; that is, the proinflammatory phenotype did not mediate the unmet needs and bachelor’s degree interaction associated with MetS. Perhaps it takes time for associations to emerge between dysregulated inflammatory signaling and cardiometabolic health, or perhaps mediation would appear with different cardiometabolic outcomes, such as atherosclerotic plaque development or symptomatic cardiovascular disease. It is also possible that associations with MetS are linked to other pathways, such as unhealthy eating behaviors.

### Strengths and Limitations

Strengths of this study include the focus on an understudied and marginalized population, the prospective design including a 20-year follow-up of families starting when children were 11 years of age, with measures during the teenage years of mental and physical health, and the investigation of cellular mechanisms in addition to tracking physical health into the adult years. Limitations include the fact that the specific outcomes of MetS and immunologic processes were not assessed during adolescence. Continuing to track the emergence of chronic diseases in this sample through the adult years will be important. In addition, comparisons with other racial and ethnic groups were not available. Furthermore, because this study did not manipulate upward mobility, we cannot draw conclusions about causality. Additionally, study attrition may have limited the generalizability of conclusions.

## Conclusions

In this 20-year longitudinal cohort study of 329 Black youths, among those from low-SES households, graduating from college was associated with an adult profile of better mental health and less substance use but worse immunologic profiles and greater risk of MetS. In contrast, among those with high-SES, graduating from college was associated with better mental and physical health in adulthood. Mechanistically, proinflammatory phenotypes (characterized by immune cells mounting larger cytokine responses to bacterial challenge) also followed a skin-deep resilience pattern. Together with other literature,^4,5^ these findings suggest the need for interventions earlier in life aimed at addressing the health profiles of upward mobility, particularly among Black youths. For example, educational interventions that aim to increase student motivation and grit^51,52^ may need to be modified to incorporate consideration of physical health as well. Interventions that consider youths holistically and focus on well-being across multiple life domains may help upward mobility become the key to positive life outcomes that we have always envisioned it to be in our society.

## Notes

Supplement 1.
eMethods

eReferences
**eTable.** Comparisons of Analytic Sample With Participants Excluded Because of Missing Data at Age 31 (at 20 Year Follow-Up)

Supplement 2.
Data Sharing Statement
