# Supplementary material for: Family Disadvantage, Education, and Health Outcomes Among Black Youths Over a 20-Year Period
Source: JAMA Netw Open. 2024 Mar 29;7(3):e242289. doi: 10.1001/jamanetworkopen.2024.2289 (PMC10980964; doi:10.1001/jamanetworkopen.2024.2289)
Supplement: Supplement 1. — eMethods eReferences eTable. Comparisons of Analytic Sample With Participants Excluded Because of Missing Data at Age 31 (at 20 Year Follow-Up) [file jamanetwopen-e242289-s001.pdf]

Supplemental Online Content

Chen E, Yu T, Ehrlich KB, et al. Family disadvantage, education, and health outcomes among black youth over a 20-year period. *JAMA Netw Open*. 2024;7(3):e242289. doi:10.1001/jamanetworkopen.2024.2289

eMethods

eReferences

eTable. Comparisons of Analytic Sample With Participants Excluded Because of Missing Data at Age 31 (at 20 Year Follow-Up)

This supplemental material has been provided by the authors to give readers additional information about their work.

© 2024 Chen E et al. *JAMA Network Open.*

eMETHODS

Participants

Data for this study were drawn from the Strong African American Healthy Adults Program (SHAPE[1]). Starting in 2001, SHAPE enrolled 667 Black children in fifth grade (mean age = 11.2 years; range 11 to 13 years) and each child's primary caregiver. Caregivers identified their children as Black. Families resided in nine rural counties of Georgia, where poverty rates are among the highest in the nation. Economically, the households of participants in this study can be characterized as working poor. Primary caregivers had a median household income of $1612 per month; 42.3% lived below federal poverty thresholds. This study was originally designed to fill a void in public health science regarding risk and protective factors among Black children residing in rural southern communities. In 2009-2010, when the youth reached age 19, a subgroup of 500 was randomly selected for a substudy of stress hormones and blood pressure. In 2021-2022, at a 20-year follow-up, when participants were on average 31 years old, we re-assessed the substudy cohort and obtained blood samples from 346 participants, from which MetS and indicators of a pro-inflammatory phenotype were assayed. The analytic sample in this study consisted of 329 individuals selected from this subsample. From the 346, one participant had missing data on unmet material needs and two others had missing data on bachelor's degree completion. The other 14 participants were not included because of technical problems with immune assessments (see below for details). The analytic sample did not differ from those in the n=500 subsample who were missing data, except for having a higher percentage of female participants (eTable 1). The University of Georgia's Institutional Review

© 2024 Chen E et al. *JAMA Network Open.*

Board approved the protocol, and written consent was obtained from participants and their caregivers at all assessments.

Data Collection Procedures

All data at all timepoints (with the exception of mental health outcomes at age 31) were collected in participants' homes through interviews and computerized surveys. A Black field researcher visited families' homes to administer computer-based interviews, allowing respondents to answer sensitive questions privately. This field researcher, who was a certified phlebotomist, also drew a fasting blood sample and collected physical health measures on participants at age 31. Age 31 mental health questionnaires were completed online.

Measures

Family Disadvantage (Ages 16-18)

When participants were 16, 17, and 18 years of age, parents completed a 4-item scale of Unmet Material Needs [2], indicating agreement (*1 = strongly agree* and *4 = strongly disagree*) with statements like "My family has enough money to afford the kind of home, clothing, food, and medical care we need." Correlations across waves ranged from .52-.58 (p's<.001), and alphas across waves ranged from .88 to .89. The three waves of assessments were averaged to form the unmet material needs composite. Higher scores indicate greater family economic disadvantage, or families having greater unmet material needs.

Bachelor's Degree Completion

At the age 31 follow-up, participants reported whether or not they had completed a bachelor's degree (n = 66, 20% with bachelor's degree).

Mental Health Composite (Age 16 and Age 31)

© 2024 Chen E et al. *JAMA Network Open.*

Because this longitudinal study spanned from the pre-adolescent years to the adult years, mental health measures were changed over time, so that age-appropriate standardized measures could be taken at each data collection time point. In order to investigate changes over time in study analyses, we compiled composite mental health measures of similar constructs at age 16 and at age 31, and employed an analytic procedure utilizing residualized change scores (described in detail in the Statistical Approach section).

At age 16, a mental health composite was formed from measures of anxiety, depression, anger, aggressive behaviors, and impulsivity. For anxiety, parents completed the 13-item Anxious/Depressed subscale of the Child Behavior Checklist about their child participating in the study[3]. Each item was rated on a Likert-type scale ranging from 0 (not true) to 2 (very true or often true). Example items include, "My child is nervous, high-strung, or tense" and "My child feels fearful or anxious." Cronbach's alpha was .78. Higher scores indicate greater anxiety. Depressive symptoms were measured via youth responses on the Child Depression Inventory[4]. Each item was rated on a Likert-type scale ranging from 0 to 2. Example items include, "In the past two weeks I felt: 0 = sad once in a while to 2 = sad all the time; and 0 = I did most things ok to 2 = I did everything wrong." Cronbach's alpha was .84. Higher scores indicate greater depressive symptoms. Anger was measured using the 15-item Anger Subscale from the State-Trait Anger Expression Inventory[5]. Youth were asked about their feelings over the past 3 months and to rate discrete emotions (e.g., "I am furious"; "I feel angry") on a scale ranging from 1 (never) to 5 (always). Cronbach's alpha was .91. Higher scores indicate greater anger. Aggressive behaviors were measured using the 17-item Aggressive Behavior subscale from the Youth Self-Report Scale (YSR)[3]. Each item was rated on a Likert-type scale ranging from 0 (not

© 2024 Chen E et al. *JAMA Network Open.*

true) to 2 (very true or often true). Example items include, “I get in many fights” and “I threaten to hurt people.” Cronbach’s alpha was .86. Higher scores indicate more aggressive behaviors. Impulsivity was measured using the 13-item Impulsiveness Subscale taken from Eysenck’s Impulsivity Inventory[6]. Each item was rated on a Likert-type scale ranging from 1 (not at all true) to 5 (very true). Example items include, “I often do things without stopping to think” and “I often get into trouble because I do things without thinking.” Cronbach’s alpha was .86. Higher scores indicate greater impulsiveness. The five indicators of mental health problems at age 16 were standardized and summed to form an age 16 mental health composite (*r*s ranged from .21 to .61, $p < .001$).

At age 31, a mental health composite was formed from adult measures of anxiety, depression, anger, aggressive behaviors, and emotional reactivity. Anxiety was measured using the 7-item Anxiety Scale [7]. Each item was rated on a Likert-type scale ranging from 1 (strongly disagree) to 5 (strongly agree). Example items include, “I feel anxious or nervous” and “I feel tense or keyed up.” Cronbach’s alpha was .89. Higher scores indicate greater anxiety. Depressive symptoms were measured using the 20-item CES-Depression Scale[8]. Each item was rated on a Likert-type scale ranging from 0 (rarely or none of the time) to 3 (most or all the time). Example items include, “How often do you feel sad” and “How often do you feel like your life is a failure.” Cronbach’s alpha was .87. Higher scores indicate greater depressive symptoms. Anger was measured by the 8-item Anger/Hostility scale [7]. Each item was rated on a Likert-type scale ranging from 1 (*strongly disagree*) to 5 (*strongly agree*). Example items include, “You feel a lot of anger inside you” and “You get mad at other people easily.” Cronbach’s alpha was .84. Higher scores indicate greater anger. Aggressive behaviors were

© 2024 Chen E et al. *JAMA Network Open.*

measured using the 15-item Aggressive Behavior subscale from the Adult Self-Report Scale (ASR[9]). Each item was rated on a Likert-type scale ranging from 0 (*not true*) to 2 (*very true or often true*). Example items include, "I get in many fights" and "I threaten to hurt people." Cronbach's alpha was .86. Higher scores indicate more aggressive behaviors. Emotional reactivity was measured using the 6-item Emotion Reactivity Subscale from the MacArthur Reactive Responding Scale [10]. Each item was rated on a Likert-type scale ranging from 1 (*strongly disagree*) to 5 (*strongly agree*). Example items include, "I often respond quickly and emotionally when something happens" and "Sometimes I overact to situations." Cronbach's alpha was .80. Higher scores indicate more emotional reactivity. The five indicators of mental health problems at age 31 were standardized and summed to form an age 31 mental health composite (*r*s ranged from .47 to .68, $p < .001$).

Substance Use Composite

At age 16 and 31, participants reported their past-month cigarette, alcohol, and marijuana use on a widely used instrument from the Monitoring the Future Study[11]. A 7-point response set ranging from not at all to more than two packs a day was used for cigarette use; a 6-point scale ranging from none to 20 or more times was used for alcohol and marijuana use. Responses to these items were summed to form a substance use composite, a procedure that is consistent with prior research[12]. Past-month substance use composite was log-transformed because its distribution was skewed.

Metabolic Syndrome Diagnosis

At the age 31 assessment, a phlebotomist visited each participant's home in the morning to draw an overnight fasting blood sample. Blood was drawn into serum separator

tubes (Becton, Dickinson and Company, Franklin Lakes, NJ). Specimens were centrifuged on site at 1500 × g for 20 minutes. The serum was harvested, divided into aliquots, and immediately frozen on dry ice. Upon arrival at the local hospital lab, it was placed in storage at -80 C until the end of the project. Glucose, high-density lipoproteins (HDL), and triglycerides were measured on a Beckman Coulter AU5800 analyzer (Beckman Coulter, Brea, CA). The average intra- and inter-assay coefficients of variation for glucose were 0.4% and 0.7%, respectively. The average intra- and inter-assay coefficients of variation for HDL and triglycerides were below 0.6% and 2.4%, respectively. Resting blood pressure was monitored with a Critikon Dinamap Pro 100 (Critikon; Tampa, FL) while the participant sat reading quietly. Three readings were taken every 2 minutes, and the average of the last two readings was used as the resting index. The field researcher measured waist circumference twice at the midpoint of the upper iliac crest and lower costal margin, at the midaxillary line. If readings differed by 1 cm, they were repeated, and the closest two values averaged.

MetS was diagnosed according to the International Diabetes Federation guidelines [13]. These criteria specify that in adults, a MetS diagnosis requires central adiposity, which for the Black participants in this sample, is defined as waist circumference ≥ 94 cm for males and ≥ 80 cm for females. At least two of four additional components must also be present. They include (a) signs of early hypertension (systolic pressure ≥ 130 or diastolic pressure ≥ 85), (b) elevated triglycerides (>150 mg/dL), (c) raised fasting glucose (≥ 100 mg/dL), or (d) lowered high-density lipoprotein levels (< 40 mg/dL in men and < 50 mg/dL in women). 38% of participants met criteria for metabolic syndrome diagnosis.

Pro-inflammatory Phenotype

© 2024 Chen E et al. *JAMA Network Open.*

Inflammation that becomes dysregulated and chronic is considered a key driver of numerous chronic degenerative diseases[14–16]. Because this study involves a longitudinal investigation of initially healthy Black pre-adolescents, measures of inflammatory processes were included as a potential early warning sign of chronic diseases later in life. Mechanistically, experiences of stress early in life are known to calibrate the response tendencies of innate immune cells so they mount excessive inflammatory responses to microbial challenges and become insensitive to glucocorticoid hormones that regulate these responses [17, 18]. This response tendency has been termed a 'pro-inflammatory phenotype,' and can lead to a chronic state of low-grade inflammation in the body, a profile that is linked to future risks of diabetes, cardiovascular disease, and cancer [18–22].

To quantify features of the pro-inflammatory phenotype, we used a portable cell-culture protocol developed for field settings [23]. In this protocol, immune cells are incubated *ex vivo* with a bacterial product (LPS, or lipopolysaccharide), and production of pro-inflammatory cytokines is measured. Hydrocortisone is added to separate cultures alongside LPS; because this compound inhibits cytokine production, it reflects how sensitive cells are to the anti-inflammatory properties of glucocorticoids.

Phlebotomists drew 6-ml of fasting antecubital blood into a Sodium Heparin Vacutainer (Becton-Dickinson). 250 ul of blood was dispensed into microfuge tubes containing LPS (diluted in R10 to an in-well concentration of 50 ng/ml) and hydrocortisone (diluted in R10 to in-well concentrations of either 10^{-6}, 10^{-5}, or 0 nM). A negative control was also prepared to measure nonspecific cytokine production, where 250 ul of blood was added to a microfuge tube with 97 ul of R10. In all conditions, the final in-well dilution of blood was 72% v/v. The samples were

© 2024 Chen E et al. *JAMA Network Open.*

incubated in a portable device for 6 hours at 37°C (Embryonentransp Model 19180; Minitube GmbH), after which supernatants were harvested by centrifugation and frozen at -80°C. When the study was completed, the samples were thawed and diluted 42-fold with diluent supplied by the assay manufacturer (Simple Plex, Protein Simple). Three inflammatory cytokines were then measured – interleukin (IL)-1β, IL-6, and tumor necrosis factor (TNF)-α – with an automated microfluidic platform (Simple Plex, Protein Simple [24]). Each cytokine was measured in triplicate by a technician blind to other participant data. Across runs, the average intra-assay coefficients of variation for triplicate samples were 2.41% for IL-6, 3.67% for IL-1β, and 1.91% for TNF-α. The inter-assay CV's were 6.24% for IL-6, 8.23% for IL-1β, and 5.37% for TNF-α.

These assays yielded a large volume of data. With 3 cytokines measured in 4 conditions, 12 outcome variables were produced. To alleviate concerns about false discovery, we decided a priori to conduct primary analyses on two composite endpoints. The first was a *stimulated cytokine production composite*, formed by averaging z-scored values of the three cytokines in the LPS well, which were strongly inter-correlated (Spearman *r*'s from 69-.78). The composite was internally consistent (Cronbach's α=.85) and scored so that higher values represent larger cytokine responses to LPS. The second was a *sensitivity to glucocorticoid inhibition composite*. It was formed by estimating participant-specific sensitivity slopes for each cytokine [17, 25]. Z-scored values of the cytokine slopes were then averaged, given the pattern of strong inter-correlation among them (Spearman *r*'s from 66-.74). Again, the composite was internally consistent (α=.91) and scored so that higher values represent greater sensitivity to the anti-inflammatory properties of glucocorticoids. Before the composites were calculated, raw cytokines values were natural log transformed to correct for skewness in their distribution. To adjust for

© 2024 Chen E et al. *JAMA Network Open.*

nonspecific cytokine production, values from the negative control well - with R10 alone – were residualized from cytokines produced in the LPS condition. 14 participants had excessive cytokine production in the negative control well, defined as more than 2 SD above the sample mean. Because these values could reflect contamination, incubator failures, or other technical problems, we excluded these participants from analysis.

Covariates

Child gender was dummy coded; male participants were coded 1 and female participants were coded 0. The SHAPE cohort was initially recruited for a randomized controlled trial of a family-oriented intervention to prevent youth behavior problems and substance abuse. Participation in the intervention was not associated with study outcomes. To minimize any residual confounding, however, we included a dichotomous covariate reflecting intervention condition (treatment vs. control) in all models.

Statistical Approach

Data analyses were completed in 2023. Study hypotheses were tested using linear regression equations (logistic regression for MetS) with sequentially entered blocks of variables: (a) covariates of gender and intervention status; (b) unmet material needs and bachelor's degree completion; and (c) a two-way interaction term of unmet material needs × bachelor's degree completion. Outcome variables included age 31 mental health composite, substance use composite, pro-inflammatory phenotype, and MetS. Interaction analyses were conducted according to established guidelines[26].

Given the longitudinal nature of the study, we tested whether the above variables were also associated with change in mental health and substance use outcomes over time. Changes

© 2024 Chen E et al. *JAMA Network Open.*

in mental health and substance use composites from age 16 to age 31 were computed as residual scores using a regression procedure in which age 31 mental health and substance use composites were regressed on age 16 mental health and substance use composites. The residualized change score is the difference between the observed outcome scores at age 31 and the predicted outcome score from the age 16 composite[27]. A positive value on the residualized change scores indicates increases in mental health problems and substance use over time, whereas a negative value indicates decreases in these outcomes over time. The residualized change score is the appropriate approach when using composite scores and standardized variables because of variables not being identical over time[27].

For physical health measures, we were not able to calculate change scores over time since there was no measure of MetS or pro-inflammatory phenotype taken during childhood/adolescence. Instead, for these analyses, age 19 BMI was statically controlled to test whether findings held after including earlier health as a covariate.

Mediated moderation analyses were conducted using a regression-based procedure. The regression coefficients were calculated for the associations of predictor (interaction between unmet material needs × bachelor's degree completion) and mediators (stimulated cytokine production or sensitivity to glucocorticoids) as path A, and for the associations of mediators and the outcome (MetS diagnosis) as path B. The indirect effect (mediation) was quantified as the product of the two regression coefficients (A x B).

All statistical tests were 2-tailed with alpha set to 0.05.

© 2024 Chen E et al. *JAMA Network Open.*

eReferences

1. Brody GH, Yu T, Chen E, Miller GE, Kogan SM, Beach SRH. Is resilience only skin deep? Rural African Americans' preadolescent socioeconomic status-related risk and competence and age 19 psychological adjustment and allostatic load. *Psychological Science*. 2013;24 1285-1293.

2. Conger RD, Elder GH. *Families in troubled times*. New York, NY_: Aldine de Gruyter; 1994.

3. Achenbach TM, Rescorla LA. *Manual for the ASEBA school-age forms and profiles*. Burlington, VT: University of Vermont, Research Center for Children, Youth, and Families; 2001.

4. Kovacs M. The Children's Depression Inventory (CDI). *Psychopharmacology Bulletin*. 1985;21 995-998.

5. Spielberger CD, Jacobs G, Russell SL, Crane RS. Assessment of anger: The State-Trait Anger Scale. In: Butcher JN, Spielberger CD, eds. *Advances in personality assessment*. Hillsdale, NJ : Erlbaum; 1983:159-187.

6. Eysenck SBG, Eysenck HJ. Impulsiveness and venturesomeness: Their position in a dimensional system of personality description. *Psychological Reports*. 1978;43 1247-1255.

7. Joe GW, Broome KM, Rowan-Szal GA, Simpson DD. Measuring patient attributes and engagement in treatment. *Journal of Substance Abuse Treatment*. 2002;22 183-196.

8. Radloff LS. The CES-D scale: A self-report depression scale for research in the general population_. *Journal of Applied Psychological Measurement*. 1977;1 385-401.

9. Achenbach TM, Rescorla LA. *Manual for the ASEBA adult forms and profiles*. Burlington, VT: University of Vermont, Research Center for Children, Youth, and Families; 2003.

10. Taylor SE, Seeman TE. Psychosocial resources and the SES–health relationship. *Annals of the New York Academy of Sciences*. 1999;896 210-225.

11. Johnston LD, O'Malley PM, Bachman JG, Schulenberg JE. *Monitoring the Future. National survey results on drug use, 1975-2006. Volume I: secondary school students (NIH Publication 07-6205)*. Bethesda, MD: National Institute on Drug Abuse, National Institutes of Health. Retrieved from http://monitoringthefuture.org/pubs/monographs/vol1_2006.pdf; 2007.

© 2024 Chen E et al. *JAMA Network Open.*

12. Chen E, Miller GE, Yu T, Brody GH. The Great Recession and health risks in African American youth. *Brain Behavior and Immunity*. 2016;53 234-241.

13. Cornier MA, Dabelea D, Hernandez TL et al. The metabolic syndrome. *Endocrine Reviews*. 2008;29 777-822.

14. Furman D, Campisi J, Verdin E et al. Chronic inflammation in the etiology of disease across the lifespan. *Nature Medicine*. 2019;25 1822-1832.

15. Schloss MJ, Swirski FK, Nahrendorf M. Modifiable cardiovascular risk, hematopoiesis, and innate immunity. *Circulation Research*. 2020;126 1242-1259.

16. Nathan C, Ding A. Nonresolving inflammation. *Cell*. 2010;140 (6):871-882.

17. Lam PH, Chen E, Chiang JJ, Miller GE. Socioeconomic disadvantage, chronic stress, and pro-inflammatory phenotype: An integrative data analysis across the lifecourse. *PNAS Nexus*. 2022;1 1-9.

18. Miller GE, Chen E, Parker KJ. Psychological stress in childhood and susceptibility to the chronic diseases of aging: moving toward a model of behavioral and biological mechanisms. *Psychol Bull*. 2011;137 (6):959-997.

19. Libby P, Theroux P. Pathophysiology of coronary artery disease. *Circulation*. 2005;111 (25):3481-3488.

20. Danesh J, Whincup PH, Walker MS et al. Low grade inflammation and coronary heart disease: Prospective study And updated meta-analyses. *BMJ: British Medical Journal.* 2000;321 199-204.

21. Mantovani A, Allavena P, Sica A, Balkwill F. Cancer-related inflammation. *Nature*. 2008;454 436-444.

22. Tsalamandris S, Antonopoulos AS, Oikonomou E et al. The role of inflammation in diabetes: Current concepts and future perspectives. *European Cardiology Review*. 2019;14 50-59.

23. McDade TW, Aronoff JE, Leigh AKK et al. Out of the lab and into the field: Validation of portable cell culture protocols. *Psychosomatic Medicine*. 2021;83 283-290.

24. Aldo P, Marusov G, Svancara D, David J, Mor G. Simple plex: A novel multi-analyte, automaed microfluidic immunoassay platform for the detection of human and mouse cytokines and chemokines. *American Journal of Reproductive Immunology*. 2016;75 678-693.

© 2024 Chen E et al. *JAMA Network Open.*

25. Chiang JJ, Chen E, Leigh AKK, Hoffer LC, Lam PH, Miller GE. Familism and inflammatory processes in African American, Latino, and White youth. *Health Psychology*. 2019;38 306-317.

26. Aiken LS, West SG. *Multiple regression: Testing and interpreting interactions*. London: Sage Publications; 1991.

27. Cohen P, West SG, Aiken LS. *Applied multiple regression/correlation analysis for the behavioral sciences*. New York, NY: Psychology Press; 2014.

© 2024 Chen E et al. *JAMA Network Open.*

eTable 1. Comparisons of Analytic Sample With Participants Excluded Because of Missing Data at Age 31 (at 20 Year Follow-Up)

	Analytic Sample	Cases Missing at Age 31		
	n (%) or Mean (*SD*)	n (%) or Mean (*SD*)	*χ^2 or t*	*p*
	(n = 329)	(n = 164)		
Mental health composite (age 16)	0.09 (3.70)	-0.18 (3.27)	0.79	.43
Substance use composite (age 16)	0.06 (0.16)	0.04 (0.12)	1.55	.12
	(n = 329)	(n = 173)		
Male	117 (35.6%)	111 (64.2%)	37.41	.000
Female	212 (64.4%)	62 (35.8%)		
Intervention	193 (58.7%)	97 (56.1%)	0.31	.58
Unmet material needs (ages 16-18)	9.04 (2.60)	8.79 (2.55)	1.01	.31
BMI (age 19)	28.18 (8.33)	26.74 (7.14)	1.90	.06

© 2024 Chen E et al. *JAMA Network Open.*
