# Supplementary material for: Family Disadvantage, Education, and Health Outcomes Among Black Youths Over a 20-Year Period
Source: JAMA Netw Open. 2024 Mar 29;7(3):e242289. doi: 10.1001/jamanetworkopen.2024.2289 (PMC10980964; doi:10.1001/jamanetworkopen.2024.2289)
Supplement: Supplement 2. — Data Sharing Statement [file jamanetwopen-e242289-s002.pdf]

Data Sharing Statement

Chen. Family Disadvantage, Education, and Health Outcomes Among Black Youth Over a 20-Year Period. *JAMA Netw Open*. Published March 29, 2024.
doi:10.1001/jamanetworkopen.2024.2289

Data
Data available: No
